# Crustose coralline algae increased framework and diversity on ancient coral reefs

**DOI:** 10.1371/journal.pone.0181637

**Published:** 2017-08-04

**Authors:** Anna Weiss, Rowan C. Martindale

**Affiliations:** Department of Geological Sciences, University of Texas at Austin, Austin, Texas, United States of America; Naturhistoriska riksmuseet, SWEDEN

## Abstract

Crustose coralline algae (CCA) are key producers of carbonate sediment on reefs today. Despite their importance in modern reef ecosystems, the long-term relationship of CCA with reef development has not been quantitatively assessed in the fossil record. This study includes data from 128 Cenozoic coral reefs collected from the Paleobiology Database, the Paleoreefs Database, as well as the original literature and assesses the correlation of CCA abundance with taxonomic diversity (both corals and reef dwellers) and framework of fossil coral reefs. Chi-squared tests show reef type is significantly correlated with CCA abundance and post-hoc tests indicate higher involvement of CCA is associated with stronger reef structure. Additionally, general linear models show coral reefs with higher amounts of CCA had a higher diversity of reef-dwelling organisms. These data have important implications for paleoecology as they demonstrate that CCA increased building capacity, structural integrity, and diversity of ancient coral reefs. The analyses presented here demonstrate that the function of CCA on modern coral reefs is similar to their function on Cenozoic reefs; thus, studies of ancient coral reef collapse are even more meaningful as modern analogues.

## Introduction

Crustose coralline algae (CCA) are non-geniculate, red algae of the order *Corallinales* that secrete Mg-calcite skeletons [1]. CCA are common worldwide, particularly in tropical regions, where CCA build algal ridges [2], act as free-living rhodoliths [3], and are key producers of carbonate sediment in reefs [3, 4, 5]. Since their appearance in the Early Cretaceous [6], CCA have developed an important, complicated relationship with corals. On modern coral reefs, CCA function as frame-builders and encrusting or binding organisms that stabilize reef accretion, prevent bioerosion, and induce settlement of coral and other invertebrate larvae ([4, 7, 8, 9, 10] and references therein).

The services that CCA provide to corals and reefs today have been shown to substantially impact reef type and diversity in modern ecosystems. CCA are critical for reef framework particularly in high-energy, intertidal or outer ridge habitats where wave-resistivity is important [8, 9]. A framework is an *in situ* aggregation of densely packed organisms (e.g., corals and CCA) often encrusted by secondary organisms (e.g., CCA, bryozoans, serpulids) [8], which forms a rigid structure. CCA act as important reef binders and reinforce caves and cavities, decreasing the likelihood of structural collapse [5, 8]. In many Caribbean reefs, CCA and marine cements bind together broken and *in situ* corals to create the primary reef structure [11]. While this is not primary framework formation in the strictest sense, CCA still contribute to the creation of a hard, rigid, and structurally complex substrate for reef accretion. Coral reefs with exposed CCA also maintain higher biodiversity than those covered by fleshy algae [12] and a drop in CCA abundance hinders coral recruitment [13]. Therefore, CCA are critical to modern coral reef ecosystems [3, 9].

Given their importance in modern systems, CCA are assumed to play an essential role on fossil reefs, especially in the Cenozoic Era when predominantly coral-algal reefs first appear [14, 15]. Following the end-Cretaceous extinction, the rise and proliferation of coral reefs is attributed to the proliferation of CCA [15, 16]. Nevertheless, to date, the global, long-term relationship of CCA with reef-building corals has not been quantitatively assessed. Like corals, CCA are sensitive to ocean warming, acidification, and synergistic impacts of climate change (e.g. [1]). Establishing a baseline for the evolution and importance of the CCA-coral relationship is critical to our understanding of reef evolution. Once established, this baseline provides an opportunity to ask nuanced questions about future reef response to climate change based on the ecology of these two keystone organisms [1]. Studies that attempt to more accurately describe abundance and other fitness measures of taxa from paleo-communities provide valuable data to compare the response of living assemblages to modern environmental change [17]. This paper tests the role and importance of CCA in fossil coral reefs.

## Materials and methods

### Data compilation

**Fig 1:**
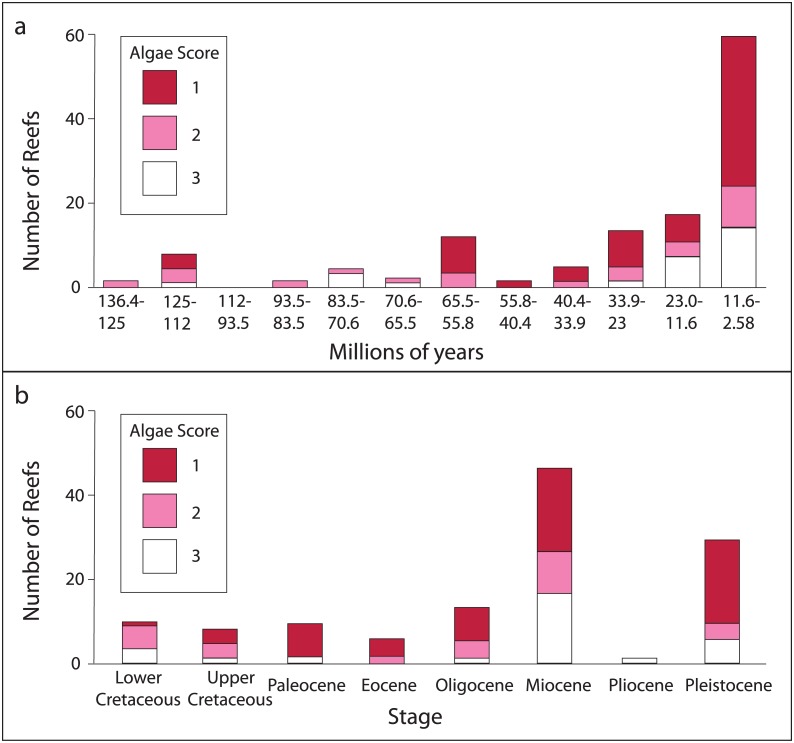
Distribution of Algae Score. Distribution of Algae Score through time, from the Late Cretaceous (136.4 Ma) through the Pleistocene (2.58 Ma). a) Time binned in roughly 10 million year intervals following [19]. b) Time binned by stage (stages determined from PARED). Algae Score 1 is red, Algae Score 2 is pink, and Algae Score 3 is white.

A database (S1 Database) of CCA occurrences on reefs (n = 128) was built in February of 2015, and updated in November 2016. A list of occurrences for the following taxa was generated from the Paleobiology database (PBDB, www.paleobiodb.org): Sporolithaceae, Hapalidiaceae, Mastophoroideae, Lithophylloideae, Metagoniolithoideae (for a list of genera, see S1 Table). CCA occurrence data was then cross-referenced with reef data from the Paleoreefs Database (PARED, www.paleo-reefs.pal.uni-erlangen.de; [18]). This allowed each CCA occurrence to be linked to a specific reef. As PARED contains far fewer post-Pleistocene entries than the PBDB, post-Pleistocene occurrences were removed from the analysis. In cases where there was no Paleoreef number associated with the CCA occurrence, the literature was consulted to check whether the CCA was reefal. Since the goal of this paper was to assess the importance of CCA on coral reefs, reefs where corals were not the primary or secondary skeletal builder were also removed. If the CCA was associated with the reef, that data point was kept. Data were binned using a) stage; and b) PBDB ‘10 million year' bins, following [19], from the Early Cretaceous (136.4–125 Ma) through Late Cenozoic (11.6–0.01 Ma). While there are differences between the data parsed by time bin (Fig 1a) versus stage (Fig 1b), in general, most reefs have at least a moderate involvement with CCA.

CCA play many roles on reefs, so simple presence/absence data is insufficient for assessing the interaction between CCA and corals. Therefore, a three-point “Algae Score” scale was developed to describe the role of CCA on coral reefs (Table 1). This Algae Score was assigned to a reef only after published references about the reef were carefully reviewed and considered. The authors took a conservative approach to building the Algae Score database, and if not enough information was provided by the literature to confidently assign an Algae Score, that data point was excluded with each data point representing one reef with a corresponding “Algae Score”. Fig 1 displays Algae Scores through time. Eighteen variables (e.g., depth, biodiversity, etc.) were collected when possible for each point (see S2 Table); herein, we focus on the most significant results, associated with diversity and reef framework building.

**Table 1:** Description of Algae Score.

Algae Score	Description
1	CCA are primary or secondary skeletal builder [8, 18]
2	CCA are of high volume or significantly binding or encrusting, but are not the main skeletal builders
3	Presence of CCA on the reef was mentioned in the literature, but nothing else was remarkable about its presence or it was otherwise noted as being a minor component

Rankings from 1 (highest involvement of CCA in reef) to 3 (CCA present but a minor or insignificant component). An Algae Score was assigned to each reef based on literature reviews and PARED and PBDB information (see text and S1 Database for details).

Data compiled from previously published works are not always comparable as different studies have distinct objectives and scopes [20]. For example, a paper may focus on the taxonomy of one reef occupant, and only briefly acknowledge other reef organisms (e.g. [21]), or fail to describe the type of bioconstruction (e.g. [22]), or focus on stratigraphy but fail to identify organisms past the Class or Order level (e.g. [23]). In some cases, multiple publications on the same reef can supply missing information, but this was not always the case. Previous database evaluations have shown that with large sample sizes (>30 to 40 [24]), broad trends and patterns are visible [25], thus the results herein, combined with the exhaustive literature review and conservative nature of data collection, are deemed robust. The database was then queried in the R programming environment (www.r-project.org), specifically RStudio; see the S1 Database for datasets.

### Statistical methods

**Fig 2:**
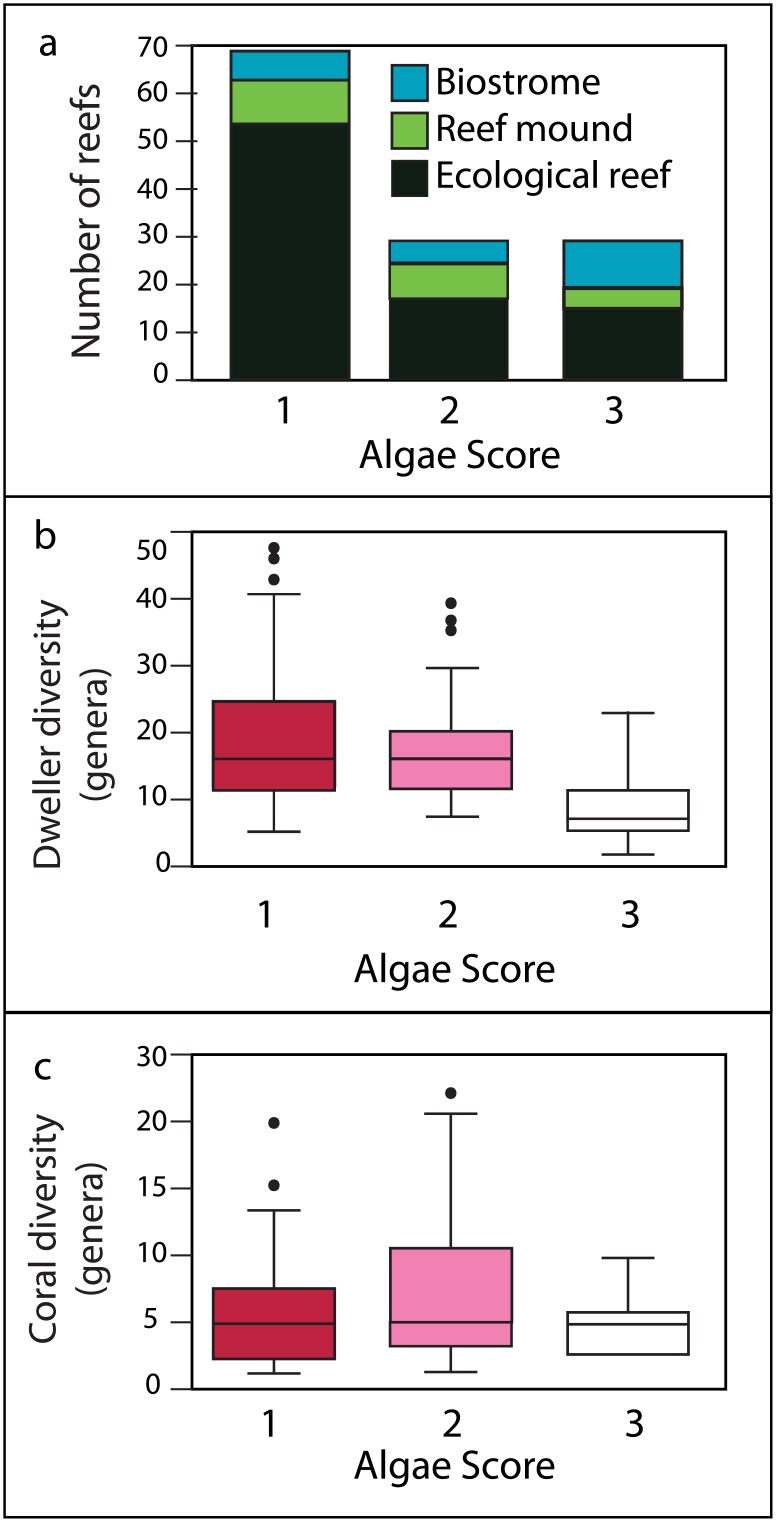
Summary of the association between reef type, diversity, and CCA abundance (all ages considered together). a) Histogram of reef type by Algae Score; definitions modified from [18]. Ecological reefs (dark green) are composed of reef building organisms that form a rigid, topographically complex structure with syndepositional relief. Reef mounds/bioherms (light green) have abundant skeletal organisms and syndepositional relief, but no clear framework; they may also contain abundant micrite and be matrix supported, although baffling can be common. Biostromes (light blue) are dense growths of skeletal organisms without syndepositional relief. Framework may or may not be present. b) Box plot showing the distribution of generic diversity for all reef dwellers, including corals. If an organism was only recorded as present, without any corresponding taxonomic information, diversity for that organism was set as equal to one. If taxonomy was only identified above the generic level, each taxon was considered once, as with genera. Reef dweller genera were recorded from the PBDB, as well as from comprehensive literature review. Algae Score 1 is red, Algae Score 2 is pink, and Algae Score 3 is white. c) Box plot showing the distribution of generic diversity for corals. If coral was only recorded as present, without corresponding taxonomic information, diversity was set as equal to one. If taxonomy was only identified above the generic level (such as family or order), each taxon was considered once, as with genera. Coral genera were recorded from the PBDB, as well as from comprehensive literature review. Color scheme follows Fig 2b.

The type of reef (e.g., ecological reef, reef mound, or biostrome) was evaluated following the terminology in the PaleoReefs database (*sensu* [18]). Ecological reefs are defined as *in situ* structures with syndepositional relief made by skeletal organisms that build a rigid framework [26], and are approximately equal to “true reefs” [18]. Reef mounds and bioherms (when broad terminology is used in the literature) have abundant skeletal organisms, with approximately an equal amount of micrite/cement, topographic structure, but no true framework; the build-up is matrix-supported and syndepositional relief is present. Biostromes are defined as dense growths of skeletal organisms, with no syndepositional relief evident; they may or may not have a rigid framework present. (Fig 2a). The association between CCA abundance and reef type (Fig 2a) was assessed with chi-squared tests of independence (S3C Table); all assumptions [27] were confirmed for this dataset (n = 128) (S3A and S3B Table). Standardized residuals were used post-hoc to assess which reef types and Algae Scores contributed most to Chi-squared value (S3D Table). To ensure consistency of results, the chi-squared test was then performed one thousand times, each time with a randomly sub-sampled dataset (n = 100, no replacement) (S3E Table).

Reef dweller alpha diversity data was not normally distributed (S1A and S1B Fig); therefore, the relationship between CCA and dweller diversity was assessed using a Kruskal-Wallis test (S4C Table) [28], the non-parametric version of an Analysis of Variance (ANOVA). Dweller diversity data show a Poisson-distribution; therefore, we also use a general-linear model (S4D Table) [29] with a Poisson dispersion to construct a model testing the hypothesis that mean diversity of Algae Scores are significantly different from one another. After the removal of outliers as identified by Residual versus Fitted plot (S1C Fig), the assumptions [24] (S1D and S1E Fig) of the general linear model were confirmed. A post-hoc Nemenyi test (S4E Table) [30] was applied to show which groups have statistically different median diversities. Coral alpha diversity data also exhibited a non-normal, Poisson distribution and so the analysis of the relationship between CCA and coral diversity (S2A–S2E Fig and S5D and S5E Table) was assessed using the same methods used for coral diversity. To ensure consistency of results for both dweller and coral diversity data, the Kruskal-Wallis test and General Linear Models were then performed one-thousand times for each variable, each time with a randomly sub-sampled dataset (n = 100, no replacement) (S4F–S4H and S5F–S5H Tables).

## Results and discussion

### Crustose coralline algae and reef framework

The chi-squared test indicates reef type is dependent upon Algae Score in Late Cretaceous and Cenozoic coral reefs (p = 0.006). When sub-sampled, 557 of 1000 tests were significant (p<0.05); however, the number of significant tests increased to 783/1000 when p<0.1. Because ecological reefs are the only constructions with definite framework, CCA presence is linked with framework and thus structural rigidity of the reef. Post-Hoc standardized residuals indicate that Algae Score 1 is most associated with ecological reefs, while the other reef types are positively associated with an Algae Score of 2 (see Table 1). Therefore, fossil coral reefs with a high involvement of CCA are associated with enhanced ability to build and maintain syndepostional relief and framework structures, consequently conferring a competitive advantage in high-energy carbonate facies. This, however, is not true for every data point with an Algae Score of 1; in fact, there are 15 entries with an Algae Score of 1, that are not considered ecological reefs. Therefore, Algae Score and reef type, while related, vary independently of each other. For example, in a Cretaceous reef of Bavaria, Germany ([31] PBDB No. 50512), CCA is considered to be one of the major skeletal builders of the reef, giving it an Algae Score of 1, yet without clear framework, the actual construction better fits the description of a reef mound.

The association between CCA and framework matches modern observations; reefs with significant CCA involvement in building, encrusting, and binding have more structural integrity than those without [5, 9]. High CCA reefs are more resistant to physical disturbances, and so can maintain their structure. In the Caribbean, even when the framework of the corals is destroyed by wave action, CCA can re-bind skeletal material [11], which results in the creation of structurally robust algal ridges [32]. The importance of the CCA as a binder rather than the primary builder in this example is similar to the description of Algae Score 2.

Reef type can also be a measure of a healthy carbonate factory. Increased carbonate productivity enhances the ability to grow vertically, in a topographically complex way, with robust framework [33], and tends to indicate “healthy” reefs [34]. An ecological reef will have increased carbonate productivity, compared to a reef mound, which does not produce framework *sensu stricto*, or a biostrome, which does not produce the magnitude of carbonate of an ecological reef. When ecological reefs degrade, they can transition into reef mounds, biostromes, or become overgrown with soft algae or microbes [17, 35, 36]. We conclude that, as in the modern, CCA were key components in the structural formation of ecological corals reefs in the Late Cretaceous and Cenozoic.

### Crustose coralline algae and reef biodiversity

Reef dweller diversity, i.e. total biodiversity of the reef, including corals, algae, echinoids, foraminifera, etc., is significantly different for each Algae Score (Fig 2b). A Kruskal-Wallis test was significant (p-value = 1.12e-07) as was the general linear model (p = 1.86e-7). When the data was sub-sampled, the Kruskal-Wallis test, the Gaussian-dispersed General Linear Model, and the Poisson-dispersed General Linear Model were significant (p<0.05) all 1000 runs. Box plot and post-hoc Nemenyi test reveal that reefs with Algae Scores 1 and 2 show significantly higher dweller diversity than reefs with Algae Score 3, with Algae Score 1 having the highest biodiversity (Fig 2b).

CCA take an active role in promoting reef biodiversity in several ways. By contributing to framework and binding reef sediments [4, 8], CCA help to maintain important niches; reef cavities supported by CCA are often inhabited by important cryptic communities, such as suspension feeders (e.g. bryozoans and sponges), fishes and crustaceans [8, 9]. CCA are important primary producers on reefs as well, acting as food for members of the reef community such chitons, echinoids, and parrot fish [9, 12, 37]. CCA can also directly impact biodiversity by inducing the settlement of larvae other than corals, including abalone and echinoids [38].

Coral diversity (Fig 2c) showed significant differences by Algae Score for the Kruskal-Wallis test (p = 0.002) and general linear model (p = 2.57e-05); however, unlike the other parameters, the significance of this parameter is sensitive to the addition or removal of a few data points and so is treated as unreliable and should be interpreted with caution. When the data was sub-sampled, the Kruskal-Wallis test was significant (p<0.05) 976/1000 times, the Gaussian-dispersed General Linear Model was significant 995/1000 times, and the Poisson-dispersed General Linear Model was significant all 1000 runs. Furthermore, a box plot (Fig 2c) and post-hoc Nemenyi test reveals that reefs with Algae Score 2 have a significantly higher diversity of corals than reefs with Algae Scores 1 and 3, indicating that greater involvement of CCA does not correspond to higher coral diversity. Two reasons are proposed to explain this trend; first, because coral larvae show settlement preferences in response to specific CCA taxa [39], CCA abundance may influence coral abundance rather than coral diversity (e.g. one type of CCA would only attract a few coral species). To test whether coral diversity is impacted by CCA, a more appropriate hypothesis would be: high CCA diversity correlates with high coral diversity. That said, CCA taxonomy in the fossil record is exceptionally poor [40], making it difficult to confidently test this hypothesis. In addition, increased CCA can negatively impact coral success through overgrowth and smothering [37, 41]. This further complicates the direct relationship between CCA abundance and coral diversity. A more appropriate measure of reef health would be total biodiversity (as reported above), which accounts for all contributors to the ecological niches of the reef. Today, healthy coral reefs are typically associated with high dweller biodiversity [7].

### Coral reefs, crustose coralline algae, and analogues for future reef collapse

The implications of the results presented here are significant to interpretations of fossil coral reefs and predictions for future reefs. CCA are vital members of the coral reef community and have been since the lower-mid Cretaceous (Fig 1, [6]), conferring traits that are typically associated with reef health: high biodiversity and the ability to build a strong framework. Given the physiological intolerance of CCA to perturbations such as elevated temperatures or ocean acidification [3], which have been shown to also directly impact reef framework [38], the forecast for reefal coralline algae looks poor [3]. Significant climate change would not only affect corals, but also the CCA, which are key reef constituents. For example, a decrease in the abundance of CCA as structural binders will impact the structural integrity of the reef [42, 43].

An important next step will be to study the relationship between CCA and reef health in detail during past climate perturbations (e.g. the Paleocene-Eocene Thermal Maximum or Pleistocene deglaciation) to assess specifically how this relationship is affected by climate events similar to those predicted for the near future. As can be seen in Fig 1, several time slices, binned both by 10 million years and Stages, have very few data points in them, making it difficult to quantitatively evaluate these trends through time [18]. For such questions, database analyses often fall short, and site-specific studies can provide more meaningful, high-resolution data [44], particularly for short-term perturbations such as the Middle Eocene Climatic Optimum or Mid-Miocene Climatic Optimum [45, 46, 47], whose short-lived durations are lost in the coarseness of the time bins [44]. Further, many of the data points have poor age control and are only resolved to the stage level, rather than sub-stage. Previous database evaluations have shown that when age control is poor, statistical analysis of data at the stage level is unwise [18]. Until more data are collected and age assignments for these reefs become more precise, the best that can currently be done is to visualize the distributions of Algae Score through time (Fig 1a and 1b). That said, we can qualitatively evaluate whether the relationship of CCA and coral reefs was impacted by past climate change events.

Given what is known about CCA sensitivity to ocean acidification and heat stress, time periods where these stressors are present will be ideal case studies. One good example is the Paleocene-Eocene Thermal Maximum, a carbon cycle perturbation event 56 million years ago for which there is evidence of rapid global warming [45, 46] and surface ocean acidification [48]. During the late Paleocene, coral reef volume was drastically reduced, disappearing in many places globally [49]; however, reefs and other bioconstructions were built by organisms such as foraminifera and microbes, and bound in part by coralline red algae [49]. This event represents a decoupling between the history of reefs, corals, and red algae, and suggests that reef collapse at this time is not due to the inability of CCA to build and strengthen framework.

Ancient analogues for modern coral reef change can be particularly useful for assessing regional and global patterns of migration in response to past environmental change [17, 50, 51] and the responses of individual taxa to climate change, which can provide information on the timing of response to climate change and extinction vulnerability [17, 52]. Nevertheless, for these deep-time case studies, care must be taken to ensure that the timescales of change are comparable (or at least similar) to modern change in order to serve as good analogues [17, 53].

## Conclusions

The role of crustose coralline algae (CCA) on fossil reefs is critical to paleoecological interpretations of coral reefs. The quantitative analysis presented here demonstrates a strong association between CCA and reef diversity as well as reef building capacity in fossil coral reefs. The significance of this association is similar to observations on modern reefs; CCA are key ecosystem engineers on coral reefs, providing protection, structural support, and enhancing biodiversity in the reef ecosystems. Without CCA, coral reefs risk shifts to alternative (non-coral) stable states or even complete collapse. The results presented herein confirm that Cenozoic coral reefs had similar ecologies to modern coral reefs, and underscores the potential for past reef collapse as analogues for current and future reef stresses. Further studies of the relationship between CCA and coral reefs—and the conditions under which this relationship persists or breaks down—are of paramount importance for the conservation about modern reefs in current and future climate change.

## Supporting information

S1 Database CCA database. (XLS)

S1 Table Genera of crustose coralline algae included in the study. (XLSX)

S2 Table Variables collected for the Algae Score database. (XLSX)

S3 Table Results of significance tests for statistical analysis of CCA contribution to reef framework. (XLSX)

S4 Table Results of significance tests for statistical analysis of CCA contribution to reef dweller diversity. (XLSX)

S5 Table Results of significance tests for statistical analysis of CCA contribution to reef coral diversity. (XLSX)

S1 Fig Results of descriptive statistics for analysis of CCA contribution to reef dweller diversity. (PDF)

S2 Fig Results of descriptive statistics for analysis of CCA contribution to reef coral diversity. (PDF)
