# Supplementary material for: Crustose coralline algae increased framework and diversity on ancient coral reefs
Source: PLoS One. 2017 Aug 4;12(8):e0181637. doi: 10.1371/journal.pone.0181637 (PMC5544230; doi:10.1371/journal.pone.0181637)

S2 Fig. Results of descriptive statistics for analysis of CCA contribution to reef coral diversity

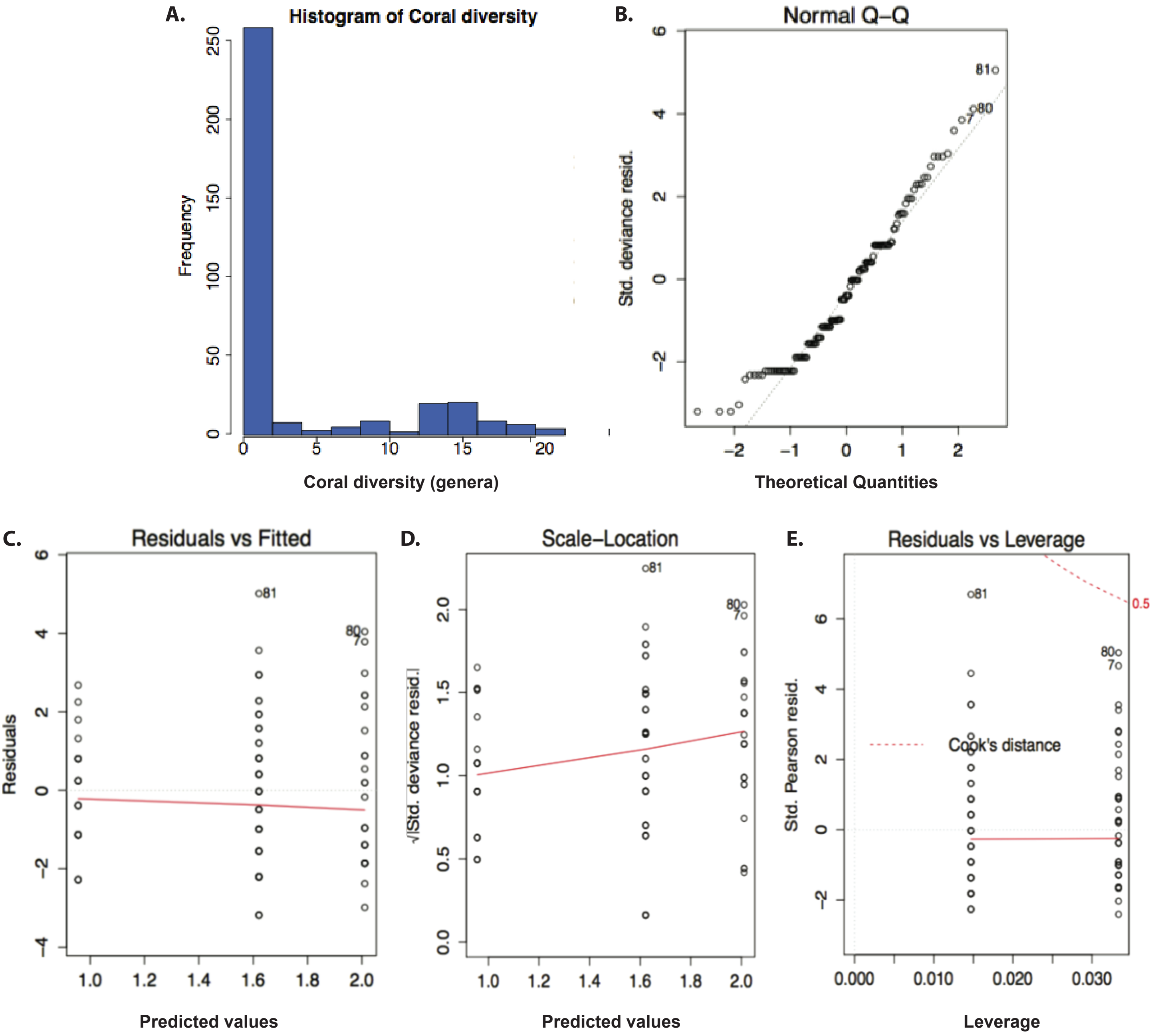

Supplement: S2 Fig — (PDF) [file pone.0181637.s008.pdf]
